# Occupational Exposure Assessment and Seroprevalence of *Brucella* Specific Antibodies Among Veterinarians in the Northern Palestine

**DOI:** 10.3389/fvets.2021.813900

**Published:** 2022-01-06

**Authors:** Ibrahim Alzuheir, Hamzeh Al Zabadi, Muhammed Abu Helal

**Affiliations:** ^1^Department of Veterinary Medicine, Faculty of Agriculture and Veterinary Medicine, An-Najah National University, Nablus, Palestine; ^2^Department of Public Health, Faculty of Medicine and Health Sciences, An-Najah National University, Nablus, Palestine; ^3^Public Health Program, Faculty of Graduate Studies, An-Najah National University, Nablus, Palestine

**Keywords:** brucellosis, ELISA, occupational exposure, Palestine, veterinarians

## Abstract

**Background:** Brucellosis is one of the most popular zoonosis in the world caused by bacteria belonging to the genus *Brucella*. The disease is considered an occupational risk to persons dealing with animals and animal products. Brucellosis is endemic in livestock in Palestine. Yet, few studies investigated human brucellosis in Palestine. We aimed to estimate *Brucella* seropositivity among veterinary healthcare professionals in Northern Palestine, and to assess the associated risk factors.

**Methods:** A cross-sectional study was conducted in four governorates in the Northern West Bank (Jenin, Nablus, Qalqylia, and Tulkarm). A sample of 100 veterinarians was collected. Participants were interviewed using a structured questionnaire to assess risk factors. Blood samples were collected to be screened for the presence of anti-*Brucella* IgG using the Enzyme-Linked Immunosorbent Assay (ELISA).

**Results:** The seroprevalence of Brucellosis by ELISA was 76%. Risk factors included working in the public sector, dealing with animals' vaccination, longer period of exposure, and advancing in age.

**Conclusions:** Brucellosis is a high-risk occupational disease among veterinarians. Its prevalence rate among veterinary healthcare workers in the Northern West Bank, Palestine was very high compared to neighboring countries and internationally.

## Background

Brucellosis is one of the most popular contagious zoonosis worldwide, caused by Gram-negative bacteria belonging to the genus *Brucella* (1). Currently, 12 recognized *Brucella* species were known, of them *Brucella melitensis, Brucella suis*, and *Brucella abortus* are the major human pathogens resulting in considerable disability and morbidity (2). The main source of human infection is contact with domestic or wild animals (3). Infected or carrier animals excrete *Brucella* through the body secretions and execrations; e.g., urine, milk, placenta, and the products of miscarriages (4). Between humans, disease transmission includes sexual contact, vertical transmission and breastfeeding (5). The infected person shows unspecified signs, such as fever, loss of appetite and weight, headache, sweating, fatigue, and back and joint pain (6). This enhances the misdiagnosis of other diseases or conditions. The gold standard and confirmatory test for brucellosis diagnosis is bacterial culture isolation and identification. However, this method is hazardous, time-consuming, and needs special infrastructure and instruments (7). Recently, serological and molecular techniques are accurate and timely reasonable for the diagnosis of human brucellosis (8). Currently, indirect enzyme-linked immunosorbent assays (ELISA) have been developed and successfully validated for diagnosis of humans and animals brucellosis (9).

On the other hand, brucellosis is endemic in humans and livestock in the developing countries of the Middle East, Asia, Africa, and the Mediterranean (10). In Palestine, there is a dramatic increase in human brucellosis in the last two decades (11). Besides the public health importance; brucellosis is considered an occupational risk for veterinary healthcare professionals, farmers, abattoir workers, and laboratory personnel (12). Therefore, this study aimed to investigate the seroprevalence rate and associated risk factors of brucellosis in veterinarians in the Northern Palestine. The results of this study might be useful to highlight the disease as a public health hazard, facilitate the control measures and the disease follow-up in different occupational professions.

## Methods

### Study Area

**Figure 1:**
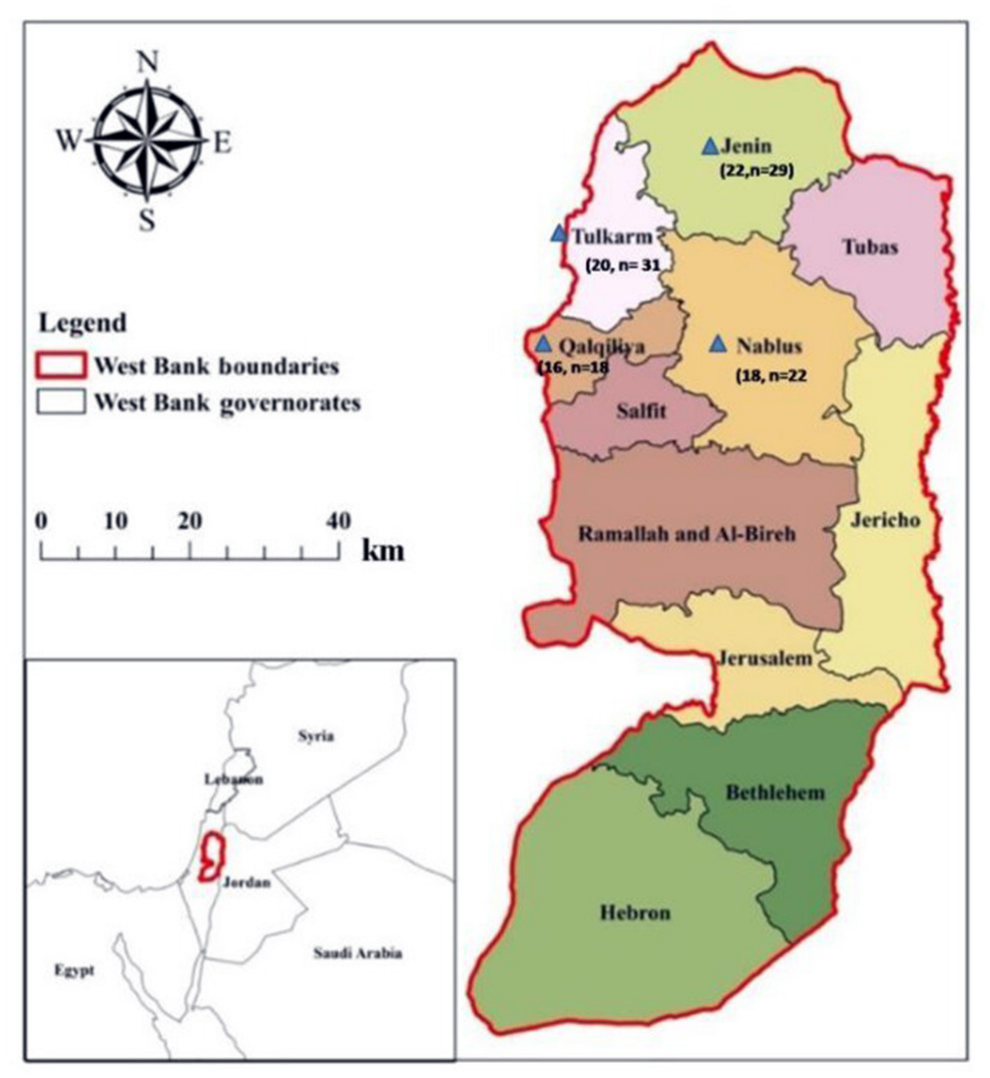
Map of Palestine. Areas where the samples were collected. Numbers indicated by a blue triangle veterinarian's collected and positive sample, respectively. Map adapted from reference (13).

The current cross-sectional study was conducted in February 2020. The study targeted the veterinarians in four cities in the Northern West Bank; Jenin, Nablus, Qalqilya, and Tulkarm (Figure 1). These cities represent 28% of the area of the West Bank and 35% of the total population (14). According to the Palestinian Veterinarians Association/Jerusalem branch, the number of practicing veterinarians in these cities was 171 out of 363 in the West Bank.

### Data Organization and Sample Collection

A questionnaire was designed to collect data regarding veterinarian location, age in years (24–29, 30–35, 36–40, 41–45, and >45), length of experience in years (1–5, 6–10, 11–15, 15–20, or >20), nature of work (public or private sector), and type of work (field or administrative). Blood samples were collected from 100 participants. After wiping with 70% ethanol, 3 ml of blood was collected from the peripheral vein in a plain tube, allowed to clot for ~2–4 h in the refrigerator. Serum was obtained by centrifugation at 3,000 rpm for 10 min at room temperature. After centrifugation, the serum was secured at −20°C until being processed.

### ELISA

Human anti- *Brucella* IgG was detected using the qualitative ELISA (Enzyme-linked Immunosorbent Assay) kit, purchased from (NovaLisa^®^ *Brucella* IgG—ELISA, IMMUNODIAGNOSTICA, Germany) and according to the manufacturer's direction. The optical density was recorded at 450 nm using an ELISA microwell plate reader. Values above cutoff were taken as positive. The sensitivity, specificity, positive predictive value (PPV) and, negative predictive value (NPV) of the test are 92.8, 79.7, 50, and 90%, respectively (15).

### Ethical Statement

The study was designed, conducted and reported in accordance with the guidelines and ethical principles of the Declaration of Helsinki for medical research involving human subjects. The experimental procedures involving human data and samples were approved by the Institutional Review Board (IRB) ethical committee at An-Najah National University with the archived number (5) November 2019. The faculty of graduate studies scientific research board council at An-Najah National University also reviewed and approved the study protocol. An explanatory sheet was attached to the questionnaire in which a full explanation of the research including the purpose, nature of the study, privacy, confidentiality, and voluntary participation was taken into account. A written-signed informed consent was obtained from each participant before participating in the study.

### Statistical Analysis

Data were analyzed with statistical software SPSS version 20th (SPSS Inc., USA). Descriptive statistics were performed and the Chi-square test was used to determine the association between the prevalence of *Brucella*-specific IgG antibodies and the other investigated factors. *P*-value < 0.05 was considered statistically significant.

## Results

In the present study, the results of a total of 100 sera samples collected from practicing veterinarians tested for the prevalence of anti-*Brucella* IgG from four cities in the Northern Palestine are shown in Table 1. There is no human brucellosis vaccine practicing in Palestine, and the seropositivity observed is caused by exposure to the bacteria or contact with the animal vaccine.

**Table 1:** Results of the indirect-ELISA brucellosis test for occupational risk factors for veterinarians working in veterinary healthcare in the Northern Palestine.

**Variable**	**Category**	**No. of** **+ve**	**No. of** **–ve**	**Total**	**Seroprevalence rate (%)**	***P*** **-value**
Location	Jenin	22	7	29	75.9	0.195
	Nablus	18	4	22	81.8	
	Qalqilya	16	2	18	88.9	
	Tulkarm	20	11	31	64.5	
Age (years)	24–29	28	12	40	70.0	0.024*
	30–35	14	11	25	56.0	
	36–40	10	0	10	100	
	41–45	4	1	5	80.0	
	>45	20	0	20	100	
Work	1–5	25	12	37	67.6	0.008*
Experience	6–10	10	0	10	100	
Length	11–15	15	0	15	100	
(years)	16–20	7	1	8	87.5	
	>20	19	1	20	95.0	
Sector	Public	32	2	34	94.1	0.010*
	Private	4	22	66	66.7	
Work nature	Field	69	20	89	77.5	0.514
	Administrative	7	4	11	63.6	

* *Statistically significant at P < 0.05*.

The overall seroprevalence rate was 76%. Anti-*Brucella* IgG was detected in all cities, age groups, work sector, work nature, and experience length. The seropositivity ranged from64.5 to 88.9%. The highest seroprevalence rate was detected in Qalqilya (88.9%, *n* = 18), and the lowest was in Tulkarm (64.5%, *n* = 31). There were no significant differences in the seroprevalence rate between the different cities (*P* = 0.195). The age of practicing veterinarians ranged from 24 to 55 years. The prevalence rate was significantly increased with the age of the veterinarian (*P* = 0.024). The seroprevalence rate was 100% in age groups 36–40 years (*n* = 10) and in >45 years (*n* = 20), 80% in age group 41–45 years (*n* = 5). The age group 24–29 years seroprevalence rate was 70% (*n* = 40), 56% in (30–35 years) age group (*n* = 25). Furthermore, the seroprevalence rate increased with the increase in years of work experience; the highest rate (100%) was observed in work experience group of 6–10 years (*n* = 37) and 11–15 years (*n* = 15). The lowest seroprevalence rate was 67.6% in the 1–5 work experience years group (*n* = 37). There was a statistically significant difference observed in the seroprevalence rate regarding work experience duration (*P* = 0.008). The seroprevalence rate in practicing veterinarians in public sector was 94.1% (*n* = 34) and was significantly higher than those in private sector 66.7% (*n* = 66; *P* = 0.01; Table 1).

## Discussion

Brucellosis is a high-risk occupational disease among veterinarians, particularly in developing countries (12). In this study, we investigated the seroprevalence brucellosis as an occupational disease in practicing veterinarians and the associated risk factors. We analyzed 100 blood samples from four cities in Northern Palestine using the ELISA test. The seroprevalence rate among practicing veterinarians in Northern Palestine was high compared to neighboring countries and worldwide. For example, in Jordan, a study investigated the prevalence of brucellosis among 66 veterinarians in different regions in the country found a rate of 43.9% (16). In Turkey, the incidence of occupational brucellosis was 11.8% (17). A cross-sectional study conducted in Hamadan, in Western Iran, between 2014 and 2015 found that the prevalence rate of brucellosis among veterinarians was 17% (18). The prevalence of brucellosis among veterinarians working in the Indian States ranges between 2.26 and 34% (19), and 44.2% among veterinarians in Ismailia-Egypt (20). The high brucellosis seroprevalence rate may be attributed to the endemic nature of the disease in livestock in Palestine (21). Other factors related to the high seroprevalence rate might include the lack of effective public health measures, inappropriate livestock disease control, the cost and availability of resources and equipment, and insufficient staff leads to a higher involvement in animal health care activities (22). It is worth mentioning that, ELISA test is a good test, but considering the PPV that might gives different false-positive results, this could in part explain the high seropositivity detected and therefore, the PPV could be a limitation of this study. However, due to high sensitivity, this kit can be used as a confirmatory diagnostic test to minimize the false-positive samples.

Our results showed that advancing in age and practicing work experience length were associated with higher brucellosis seropositivity due to longer exposure. These results agree with previous studies in Jordan (16), Egypt (23), Iran (24), and Brazil (25). Working in the public sector in Palestine was associated with higher brucellosis seropositivity. This finding is in contrast to a study in Turkey, which showed that veterinarians working in the private sector were higher brucellosis seropositivity compared to the public sector (17). Kutlu et al. (17) reported that the veterinarians in the Turkish private sector have more contact with sick animals due to privatization policies, compared to the public sector who works in preventive measures. In contrast, privatization of veterinary services policies is not applied in Palestine; and veterinarians in the public sector are responsible for dealing with livestock brucellosis cases, diagnostic facilities, and farm animals vaccinating program. Shome et al. (22) found that veterinarians who interact directly and continuously with farm animals are at a high risk of infection with brucellosis. The vaccine strain used in Palestine is Rev 1; this strain can cause asymptomatic or mild signs of human brucellosis (26). In Palestine, the vaccination against *Brucella*, collection of samples from embryos, fetal membranes, and aborted fetal fluids and laboratory diagnosis of brucellosis is restricted for the veterinarians in the public sector. This might explain that the veterinarians working in the public sector in Palestine were at risk factor for brucellosis (27). It should also be noted that the prevalence of Brucellosis among field and administrative workers were 77.5% (*n* = 69 out of 89 field workers) and 63.6% (*n* = 7 out of 11); respectively. Although filed workers had more seroprevalence rate of Brucellosis, the difference was not statistically significant as shown in Table 1 (*P*-value = 0.514). However, this information might be important and useful because administrative workers could be considered as control group with less exposure to animals.

## Conclusions

The current study showed that the seroprevalence of brucellosis among veterinarians in Northern Palestine was very high, and the disease can represent a significant occupational risk. Rising public health awareness and screening of humans and animals for brucellosis and mass vaccination measures are needed. This could be achieved through increasing the coordination between the Palestinian Ministry of Health and the Ministry of Agriculture.

## Data Availability Statement

The original contributions presented in the study are included in the article/supplementary material, further inquiries can be directed to the corresponding author.

## Ethics Statement

The study was designed, conducted, and reported in accordance with the guidelines and ethical principles of the Declaration of Helsinki for medical research involving human subjects. The study was reviewed and approved (including the experimental procedures involving human data and samples) by the Institutional Review Board (IRB) of An-Najah National University, Nablus-Palestine with an archived number of (November 5, 2019). The study was also approved by the faculty of graduate studies scientific research board council at An-Najah National University, Nablus-Palestine. A written-signed informed consent was obtained from each participant before participating in this study. The patients/participants provided their written informed consent to participate in this study.

## Author Contributions

HA, IA, and MA designed and planned the study protocol, drafted the manuscript, and participated in data analysis. MA and IA contributed to data and sample collection and ELISA work. All authors revised, designed, planned the study protocol, drafted the manuscript, participated in data analysis, and approved the final manuscript.

## Funding

The materials used in this study were provided from the Faculty of Graduate Studies at An-Najah National University (ANNU), Nablus-Palestine.

## Conflict of Interest

The authors declare that the research was conducted in the absence of any commercial or financial relationships that could be construed as a potential conflict of interest.

## Publisher's Note

All claims expressed in this article are solely those of the authors and do not necessarily represent those of their affiliated organizations, or those of the publisher, the editors and the reviewers. Any product that may be evaluated in this article, or claim that may be made by its manufacturer, is not guaranteed or endorsed by the publisher.
